# Comparative transcriptome analysis reveals molecular response to salinity stress of salt-tolerant and sensitive genotypes of *indica* rice at seedling stage

**DOI:** 10.1038/s41598-018-19984-w

**Published:** 2018-02-01

**Authors:** Jun Wang, Jinyan Zhu, Yadong Zhang, Fangjun Fan, Wenqi Li, Fangquan Wang, Weigong Zhong, Cailin Wang, Jie Yang

**Affiliations:** 1 Institute of Food Crops, Jiangsu Academy of Agricultural Sciences/Nanjing Branch of Chinese National Center for Rice Improvement, Nanjing, 210014 China; 2 Jiangsu Co-Innovation Center for Modern Production Technology of Grain Crops, Yangzhou, 225009 China

## Abstract

Abiotic stresses, such as salinity, greatly threaten the growth and productivity of plants. Rice (*Oryza sativa* L.) is one of the most important food crops, as well as a monocot model for genomic research. To obtain a global view of the molecular response to salinity stress, we conducted a leaf transcriptome analysis on rice seedlings. Two cultivars of rice subspecies *indica*, including the salt-tolerant genotype Xian156 and the salt-sensitive genotype IR28, were used in the present study. Eighteen RNA libraries were obtained from these two genotypes at three timepoints (0 h, 48 h and 72 h) after applying salinity stress. We obtained the reference-guided assembly of the rice transcriptome, which resulted in 1,375 novel genes, including 1,371 annotated genes. A comparative analysis between genotypes and time points showed 5,273 differentially expressed genes (DEGs), of which 286 DEGs were only found in the tolerant genotype. The Disease resistance response protein 206 and TIFY 10 A were differentially expressed, which were validated by quantitative real-time PCR. The differentially expressed genes identified through the mRNA transcriptome, along with the structure, provide a revealing insight into rice molecular response to salinity stress and underlie the salinity tolerance mechanism between genotypes.

## Introduction

Abiotic stress, such as low temperature, drought and salinity greatly threaten the growth and productivity of plants^1^. With the development of modern agriculture, soil salinity has become an environmental problem, and unreasonable irrigation with brackish water contributes to the aggravation of secondary soil salinity^2^. According to the FAO Land and Plant Nutrition Management Service, at least 6% of the world’s land is affected by salinity. Out of the current 230 million hectares of irrigated land, 45 million hectares are affected by salt (19.5%), and among them 1500 million hectares are under dry land agriculture, while 32 million are salt-affected to different degrees (2.1%)^3^.

Soil salinity limits plant growth initially in the form of osmotic stress and later causes ionic stress^4^. The former phase is caused by a high concentration of salt in soil that reduces water uptake by plants, which results in triggering a range of cellular and metabolic processes. Cell expansion, cell wall synthesis, protein synthesis, stomata conductance, and photosynthetic activity are all inhibited during the initial phase, whereas the accumulation of compatible solutes and abscisic acid (ABA) increased^5^. The later phase is associated with alterations in the Na^+^/K^+^ and Na^+^/Ca^2+^ ratios because of the accumulation of ions (Na^+^ and Cl^−^)^6^. Then, ion accumulation stimulates the production of reactive oxygen species (ROS). Through the additional production of ROS in cells, the balance between production and elimination of ROS is disturbed and leads to subsequent oxidative stress^7^. In addition, many studies have shown that excessive salinity in soil will inhibit the overall nutrition supply and element absorption of the plant root system^8^.

Rice (*Oryza sativa* L.), which is a Gramineae family plant that originated in Asia, is one of the most important food crops in the world. It was also favored as a model for genomic research in monocots because its genome size is relatively small. Rice is a settled plant that is unable to escape from environmental stresses. Therefore, salinity stress is a global threaten to rice productivity. The continuous intrusion of salt due to global warming will certainly hamper rice cultivation along coastal areas and other saline alkali areas. Challenges for rice production are emerging in the new century and studies have showed that salinity significantly decreased the dry matter of cultivars DM-38–88 and NR-1. Salinity also influences the yield per plant, photosynthesis, and fertility percentage^9^. Under saline conditions, metabolism of sugars is adversely affected in rice^10^. Otherwise, selection of salt tolerant cultivars is an effective method to increase the productivity of rice^9^. A correlation between the ability of salinity tolerance and grain supply in rice exists. Therefore, identifying the genes responding to salt stress greatly accelerates the study of stress tolerance mechanisms and rice breeding^11^.

With the first release of the rice draft genome^12,13^, the transcriptome sequencing and functional genomics have greatly facilitated rice research. There have been several reports aiming to determine the salt-tolerance mechanism in cultivated and wild rice. In 2010, 995 (shoot) and 1,052 (root) annotated rice genes were linked to salinity stress using thirty-six-base-pair reads produced by transcriptome sequencing of the *O. sativa* ssp. *japonica* ‘Nipponbare’^14^. Identification and analysis of novel salt responsive candidate gene based SSRs (cgSSRs) from rice (*Oryza sativa* L.) were performed. Nineteen primer sets were used to clearly detect polymorphism in diversity analysis among salt tolerant and susceptible rice genotypes^15^. After transcriptome sequencing of Dongxiang wild rice (*O. rufipogon* Griff.), 6867 transcripts were differentially expressed in the leaves (2,216 up-regulated and 4,651 down-regulated), while 4,988 transcripts in the roots were differentially expressed (3,105 up-regulated and 1,883 down-regulated). Then, a comparison with the previous RNA-Seq analysis of salt-stress responses in Nipponbare shown that unique molecular mechanisms of salt-stress responses possibly play a vital role in Dongxiang wild rice^16^. A comparative transcriptome of the Brazilian rice cultivar ‘BBRS Querência’ was conducted under various environmental stresses, including cold, iron, and salinity stresses. A total of 2,092 differentiated genes were linked to salinity stress, of which 622 genes were uniquely linked to salinity stress while another 1,470 genes also responded to three other stresses. The draft genome of *Oryza coarctata*, the only halophytic species in the genus Oryza, was available. The gene ontology analysis also identified several salt responsive genes^17^. However, determining how cultivars of the rice subspecies *indica* cope with salinity stress is unclear. In this study, we attempted to perform deep transcriptome sequencing to identify potential candidate genes with differential expression and to understand the fundamental molecular mechanisms underlying stress tolerance in rice.

## Materials and Methods

### Plant materials and RNA-seq

The seeds of the salt-tolerant rice cultivar Xian156 and the salt-sensitive cultivar IR28 were immersed in water at 37 °C for two days and then germinated at 26 °C with a photoperiod of 12 h. Seedlings were grown in 200 mL pots containing Yoshida nutrient solution in an artificial climate chamber for 14 days under controlled conditions (12 h light 30 °C/12 h dark 24 °C cycles). At the two-leaf stage, the aboveground parts of robust and uniform growing rice seedlings from these two cultivars were harvest as control samples. Then, they were grown in salinity stress condition (Yoshida nutrient solution with 0.5% NaCl solution), then they were harvested at 48 hours and 72 hours later. The 85 mM (0.5%) NaCl is an effective salinity stress concentration, which was performed in many studies^18,19^. Three replicates were used for each time point of each cultivar. Young leaves were immediately frozen in liquid nitrogen before being stored at −70 °C for further experiments.

Total RNA was extracted from these samples using the Total RNA Kit (Tiangen, Beijing, China), then the samples were concentrated using oligo (dT) magnetic adsorption and served as a template for the synthesis of first-strand cDNA using random hexamers and reverse transcriptase. Second-strand cDNA was synthesized and purified by the AMPure XP beads and resolved in EB buffer for poly(A) addition and adapter addition. The cDNA fragments with suitable lengths and insert sizes were selected by AMPure XP beads to construct the final cDNA libraries. The cDNA libraries were checked using Qubit2.0 and Agilent 2100 before they were sequenced using the Illumina HiSeq. 2500 High-throughput Sequencing machine at Genepioneer Biotechnologies Co, Ltd. (Nanjing, China). For each end of the produced paired-end sequences, 125 bases were sequenced.

### Assembly of genes and annotation

The rice genomic sequences and gene annotation were downloaded from BGI RIS (Beijing Genomics Institute Rice Information System, http://rise2.genomics.org.cn/). The clean data from all 18 libraries were separately mapped to the rice genome assembly using Tophat software (https://ccb.jhu.edu/software/tophat/index.shtml, v2.1.0)^20^. Then, the expression of all genes was determined with FPKM (Fragments Per Kilobase of exon per million fragments Mapped) values using the software Cufflinks (http://cole-trapnell-lab.github.io/cufflinks, v2.2.1)^21,22^ under the guidance of annotated gene models from a GFF file that refers to a previous report^23^.

### Alternative splicing analysis and SNP calling

To identify alternative splicing events in rice, the exon skipping, intron retention, alternative 5′ splicing, alternative 3′ splicing and the alternative final exon, the alternative splicing events were predicted by comparing mapped reads with gene models of reference genome annotations with Cufflinks software. The gene structure of alternative splicing was drawn with SpliceGrapher software^24^.

In each sequencing library, the SNP calling was performed before identifying the mismatch bases between the reference genome and sequenced reads. The mismatch was identified to as a potential SNP when it met all the following criteria: (1) the alignment score by Tophat2 was bigger than or equal to 50; (2) the nearest mismatch loci were far more than 5 bp in the genome; (3) the variation score was bigger than or equal to 20; and (4) the sequencing depth of coverage was between 5 and 100 bp.

### Novel gene identification

After aligning reads in a reference genome, paired-end reads were enriched in specific regions that were annotated to be intergenic regions using Cufflinks software. These regions were annotated to be novel genes after a short polypeptide product (length <50 amino acids) and single exon regions were eliminated. The GFF contained exon, intron and UTR regions of novel genes that were predicted according to the expression of reads. Annotations were assigned to new genes after searching against various nucleotide and protein databases. A BLASTX search was performed against the Nr, KOG, KEGG and Swiss-Prot databases using an E-value cut-off of 10^−5^. Hits with the highest sequence similarity along with their protein functional annotations were chosen to represent the most likely functions of this sequence. The Blast2GO program was used to obtain GO annotations for annotated genes through various databases. The WEGO software was then used to plot a GO functional figure for these genes with GO term hits to view the distribution of gene functions at the macro level^25^.

### Digital gene expression tag profiling

For each sample, reads were mapped into the corresponding rice gene models using bowtie (v 0.12.8)^26^. The FPKM values were chosen to measure the expression of reads. Pair-wise differential expression analysis of genes was applied between each time point in each cultivar using EBSeq software^27^. The False Discovery Rate (FDR) and log2FC (log of fold change) was calculated for all genes, and only transcripts with a fold-change ≥2 and FDR <0.1 were considered to be differentially expressed genes.

### Real-time quantitative (q)PCR validation

Three genes with a response to salinity stress identified by RNA-seq were chosen for experimental validation by qPCR, INCLUDING BGIOSGA003836, BGIOSGA021937 (Disease resistance response protein 206), and BGIOSGA031997 (*TIFY 10* *A*). The primers for these genes were designed with Beacon Designer 7 software (Table 1). qPCR assays were performed with three biological and three technical replicates. Total RNA was isolated from inoculated and control leaves using a TaKaRa MiniBEST Plant RNA Extraction Kit (9769) according to the manufacturer’s instructions. The first-strand cDNA was synthesized using M-MLV reverse transcriptase (TaKaRa, Japan). The SYBR Premix Ex Taq II reagent (Takara, Japan) with SYBR Green I as the fluorescent dye was used for qPCR on an ABI 7300 real-time PCR system (Applied Biosystems, Foster City, CA, USA). Each reaction contained 10 µL 2× SYBR Premix Ex Taq II Reagent, 1.0 µL cDNA sample, and 500 nM gene-specific primers in a final volume of 20 µL. RNA levels were expressed relative to the amount of the Actin2 gene (forward: GCCATTCTCCGTCTTGATCTTGC; reverse: AGCGACAACCTTGATCTTCATGCT)^28^ as 2^−ΔΔCt^, where Ct is the cycle threshold measured according to a previous method^29,30^.

**Table 1:** Designed primers for qPCR validation of three DEGs.

Gene	Forward Primer	Reverse Primer	Description
BGIOSGA003836	CATCGCCAAGATACTCAC	GCGTAGTAGTCCATCATG	Uncharacterized protein B1097D05.32 (Os01g0582600 protein)
BGIOSGA021937	TCACCTGCACTTCTTCAT	CAGATGTACTGCCCCTTG	Disease resistance response protein 206
BGIOSGA031997	GCTGACCATCTTCTACGA	ATCCTGTGCTTCCTCTTC	Protein TIFY 10 A

## Results

### Symptoms to salinity stress

**Figure 1:**
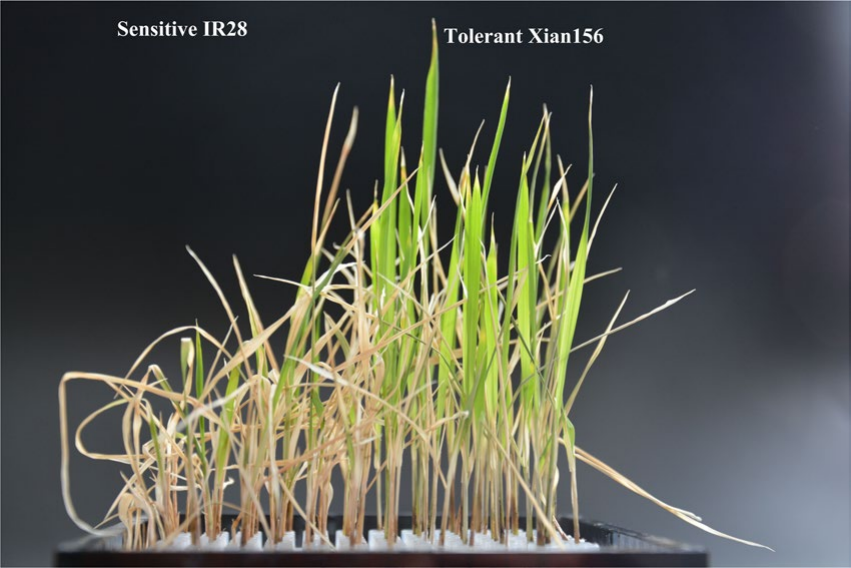
The symptoms of seedlings showed the salt tolerance of the rice sensitive IR28 genotype and the tolerant Xian156 genotype.

The salt tolerant rice genotype Xian156 was crossed by Jingang40 (female parent) and IR24 (male parent). The salt sensitive breeding line, IR28, was used in a previous report^31^. To investigate the characteristics of the molecular response to salinity stress between salt-tolerant and salt-sensitive genotypes, the Xian156 and IR28 seedlings at the two-leaf stage were exposed to the hydroponic solution with 85 mmol/L NaCl. The rice cultivar Xian156 was obtained from a cross of maternal parent Jingang30 and male parent IR24, which was close to the tolerant cultivar^32^. The salt tolerance at the seedling stage by using 0.5% NaCl solution were evaluated for rice germplasm which from regional trials of Jiangsu province and other regions in recently years. The plant height was shorten in salinity than that of normal condition and the salt tolerance is subject to influenced by environments. In general, the salt tolerance of *indica* rice is stronger than that of *japonica* rice. Based on the results of 2 years, Xian156 showed superior salt tolerance at the seedling stage. The growth vigor of these two genotypes without salt treatment was shown in (Supplementary Figure S1). At first 24 h, there was no difference in the exits between these two genotypes. After 48 h of salt treatment, both genotypes showed wilting symptoms, while the sensitive IR28 was more obvious when comparing with tolerant Xian156. At 72 h, medium wilting symptoms were shown in the tolerant Xian156 genotype, while severe wilting symptoms were shown in the sensitive IR28 genotype (Fig. 1). The time points of 0 h, 48 h and 72 h were therefore selected for characterization of gene expression to explore a possible molecular mechanism of salt-stress. The salinity tolerance and growth rate of these two genotypes was also significantly different. The dead leaf blade rate (the percentage of dead leaf blade areas in all leaf blade areas) of sensitive IR28 was 84.3%, while the leaf blade rate of tolerant Xian156 was only 38.8% at 14 d after the plant was subjected to salinity stress. The weight and height of rice seedlings before and after salinity stress were also measured. The average seedling net weight of sensitive IR28 was greatly declined to 0.3583 g at 14 d after the plant was subjected to salinity stress, when compared to 0.3583 g in control samples. In contrast, the average seedling nest weight of tolerant Xian156 was slightly decreased to 0.2717 g at 14 d after the plant was subjected to salinity stress, when compared to 0.1997 g in control samples (Supplementary Table S1).

### Overview of sequencing and transcriptome assembly

To obtain a global overview of the rice transcriptome under salinity stress, we constructed 18 cDNA libraries and performed RNA-seq, which yielded 356.98 million reads in total with 125 bp for both paired ends. After adapter removal and refining, we obtained 89.74 Gb of clean data, and Q30 of the base ratio were higher than 87.99%. The ratio of reads mapping to BGI rice genome was high, with a mapping rate fell into a range between 82.33% and 84.72% (Table 2). The saturation curve of all sequencing data from 18 RNA libraries indicated that sequencing depth was enough for transcriptome assembly and expression detection (Supplementary Figure S2).

**Table 2:** Sequencing statistics of 18 RNA libraries.

Sample	Number of Reads	Number of Bases	GC content	Q20	Q30
T01	17314331	4353934258	55.70%	93.98%	88.54%
T02	21502222	5405075871	55.58%	93.78%	88.85%
T03	20015348	5030990015	54.94%	93.91%	89.04%
T04	18696906	4700003447	55.56%	93.30%	87.99%
T05	20853371	5239716552	55.31%	93.69%	88.67%
T06	19470738	4895004244	55.19%	93.53%	88.43%
T07	20109348	5049103151	55.94%	93.59%	88.51%
T08	17677142	4443578621	56.06%	93.46%	88.31%
T09	20010359	5031525048	55.20%	93.55%	88.46%
T10	18332781	4608253840	54.33%	93.75%	88.78%
T11	18585155	4674777308	55.10%	93.58%	88.54%
T12	19783418	4969620514	54.90%	93.58%	88.53%
T13	24919531	6269627246	54.83%	93.55%	88.50%
T14	20893397	5255291187	54.42%	93.30%	88.07%
T15	19387420	4873645882	53.89%	93.82%	88.93%
T16	19225841	4835776464	55.39%	93.33%	88.16%
T17	18956297	4766495755	55.55%	93.25%	88.04%
T18	21242807	5341083919	55.95%	93.27%	88.08%
Total	356976412	89743503322	—	—	—

To assemble the rice total leaf transcriptome, the high quality filtered reads were used for reference guided transcriptome assembly, using BGI *indica* genomic sequences as the reference. For each sample, the mapped reads were assembled according to the updated reference gene models, and the expression values were calculated for each gene and each isoform separately by Cufflinks software. Other mapped reads were retained for further new gene searching, which will be discussed further in this study.

Theoretically, the sequencing reads produced from mature mRNA in cells could be mapped into exon regions with a ratio of 100%. However, a small portion of reads also be mapped onto introns and intergenic regions for the following reasons: (1) The sequencing mRNA library contained enriched mRNA, which was characterized with a Poly-A tail. Some mRNA precursors that contained both PolyA and intron regions were retained for sequencing library construction. (2) The reference genome annotation contained errors in which the exon regions and intron regions were annotated incorrectly. (3) Genes are expressed according to time-specific and tissue-specific patterns. Some coding genes and coding gene regions were possibly ignored in previous reports. (4) Variations existed between reference genome and sequencing genotypes. The SNP density in the reference genome was measured and drawn using an in-house R language script.

### Optimized Gene Structure

The prediction of gene structures, including exon, intron and UTR regions relies on the transcriptome reads mapped to the assembled genome sequences. The latest GFF files containing gene models of rice *indica* that was mainly obtained in 2005 and has been slightly modified subsequently^33^. The genomic and gene sequences were also available in the ENSEMBL website (ftp://ftp.ensemblgenomes.org/pub/plants/release-20/fasta/oryza_indica, released in 2013) under the accession ASM465v1.20. In this study, we re-aligned the reads and optimized *indica* gene models, which resulted in an updated copy of *indica* gene models that will be more representative of the transcription regions of genes under environmental stress (Supplementary Table S2).

### Identified new genes and lncRNAs by transcriptome

Although the draft genome of ‘Nipponbare’ was available in late 2002, there were dozens of studies focusing on the transcriptome of rice. However, most of them were identified and characterized in the cultivated rice subspecies *japonica*, while little information was uncovered for the subspecies *indica*. To facilitate application of rice genomic information and to provide a foundation for functional and evolutionary studies of other important cereal crops, the Beijing Genomics Institute (BGI) developed the Rice Information System (BGI RIS) to store the annotated rice *indica* genomes for gene content, repetitive elements, gene duplications (tandem and segmental) and single nucleotide polymorphisms between rice subspecies^34^, and the system has been subsequently updated several times^35^.

RNA-seq technology is an effective approach to identifying novel genes embedded in the genome, which are ignored by previous studies due to specific expression or distinct gene structures compared to well-studied genes. We identified 3,765 new transcripts in the *O. sativa* ssp. *Indica* genome, while 1,375 were annotated as coding genes. These genes were evenly distributed in all chromosomes. The exons and introns were predicted according to the reads, and then the results were shown in standard GFF format (Supplementary Table S3 and Table S4).

The non-coding RNAs were identified in these samples. To distinguish the protein-coding and non-coding potential of newly observed transcripts, we used four programs to identify potential lncRNAs. CPC, CNCI, CPAT and Pfam pipelines are four software programs to identify lncRNAs through a computational approach. Pfam is a widely used database of protein families and domains that could distinguish coding transcripts from other transcripts^36^. CPC (Coding Potential Calculator) software is a support vector machine-based classifier that can discriminate coding from noncoding transcripts with high accuracy^37^. CNCI (Coding-Non-Coding Index) software was developed to effectively classify protein-coding or non-coding transcripts by profiling adjoining nucleotide triplets independent of known annotations^38^. CPAT (Coding Potential Assessment Tool) is an alignment-free tool that can rapidly recognize coding and noncoding transcripts from a large pool of candidates using a logistic regression model built with open reading frame size, open reading frame coverage, Fickett TESTCODE statistics and hexamer usage bias^39^. The candidates obtained by these four software programs were all different. A total of 839 new genes were coding genes identified by the Pfam database, while 1032 were found to be lncRNAs by CPAT. Approximately 400 and 100 genes were predicted to be lncRNAs by CPC and CNCI, respectively, but then the same genes were predicted to have coding potential by the Pfam database. Similarly, 214 new genes were predicted to be non-coding genes by both CPC and CNCI but were predicted to be coding genes by the Pfam database (Supplementary Figure S3).

### Functional annotation of new genes

**Figure 2:**
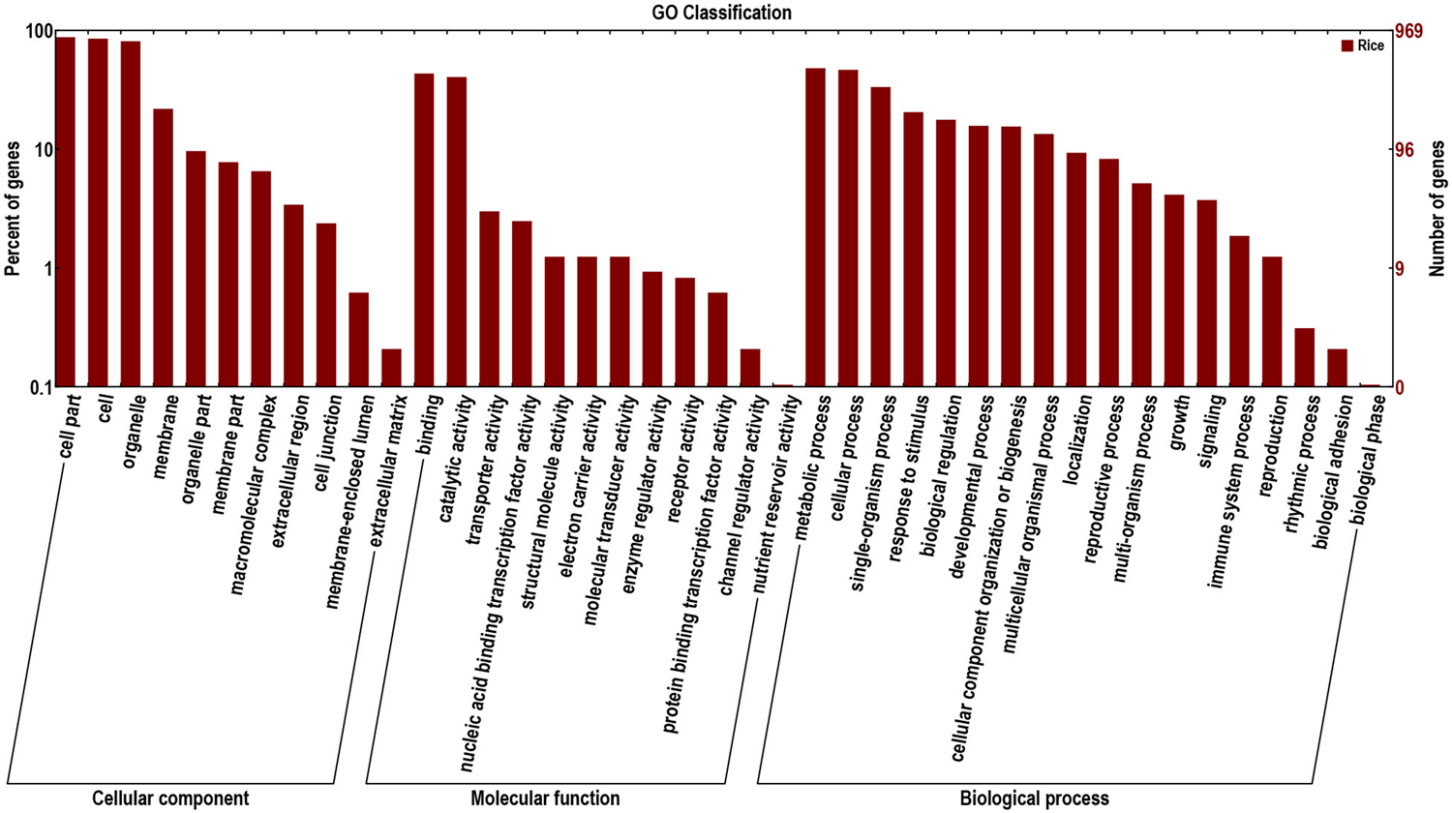
GO classification of newly identified genes in rice.

To provide insight into the functions of newly identified rice genes, these genes were annotated after they were compared to well-studied sequences in the GO, Nr, Swiss-Prot, KEGG and KOG databases. In total, 1371 out of 1375 new genes were annotated using sequences from at least one database (Supplementary Table S3). Gene Ontology (GO) enrichment analysis was carried out to provide a dynamic, controlled vocabulary and hierarchical relationships for the information on molecular functions, cellular components and biological processes^40,41^. This approach provided important access to the underlying fundamental functions of new genes. In this study, 969 newly identified genes were assigned GO terms and a hierarchical relationship picture is drawn in Fig. 2. For cellular components, the annotated genes involved in cell parts (848), cells (822) and organelles (782) were the most abundant entries. The three most common categories for biological processes were metabolic processes (464), cellular processes (449) and single-organism processes (322). For molecular functions, binding (418), catalytic activity (391) were the most two abundant catalogs. The distribution ratio considerably agreed with the ratio of GO enrichment analysis of all known rice genes.

**Figure 3:**
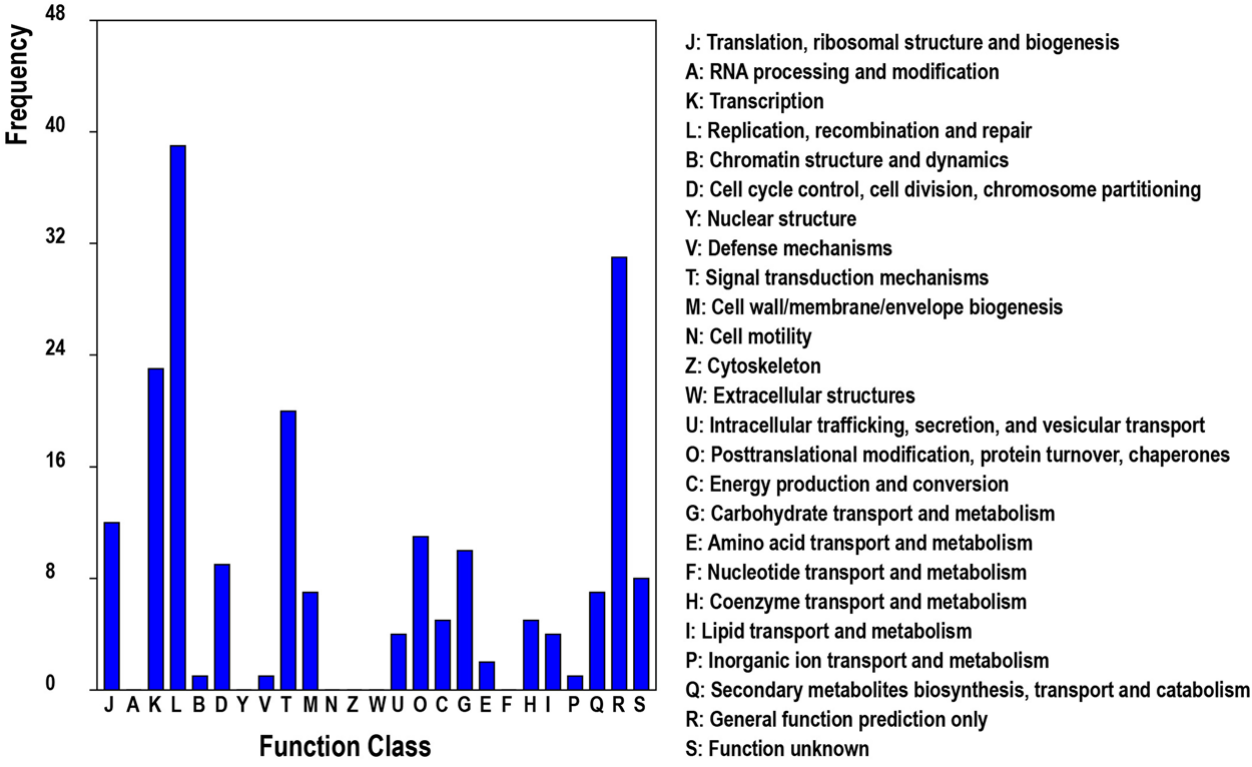
KOG classification of newly identified genes.

To better understand the biological function of these new genes, we conducted an enrichment analysis with the KEGG pathway and assigned annotations to 72 new genes. A total of 8, 6 and 4 new genes were found to participate in mRNA surveillance pathway (ko03015), RNA transport pathway (ko03013) and peroxisome pathway (ko04146). Moreover, the euKaryotic Ortholog Group (KOG) terms were assigned to 132 new genes based on the best BLASTX hit from the KOG database. Most of them were distributed in the L subgroup (Replication, recombination and repair), R subgroup (General function prediction), K subgroup (Transcription) and T subgroup (Signal transduction mechanisms) (Fig. 3). After comparing with proteins in NCBI NR database, homologs of most newly identified genes were also found in other monocots, such as *Oryza brachyantha*, *Zea mays*, *Oryza sativa* and *Setariz italic* (Supplementary Figure S4).

### Real-time quantitative PCR (qPCR) validation

**Figure 4:**
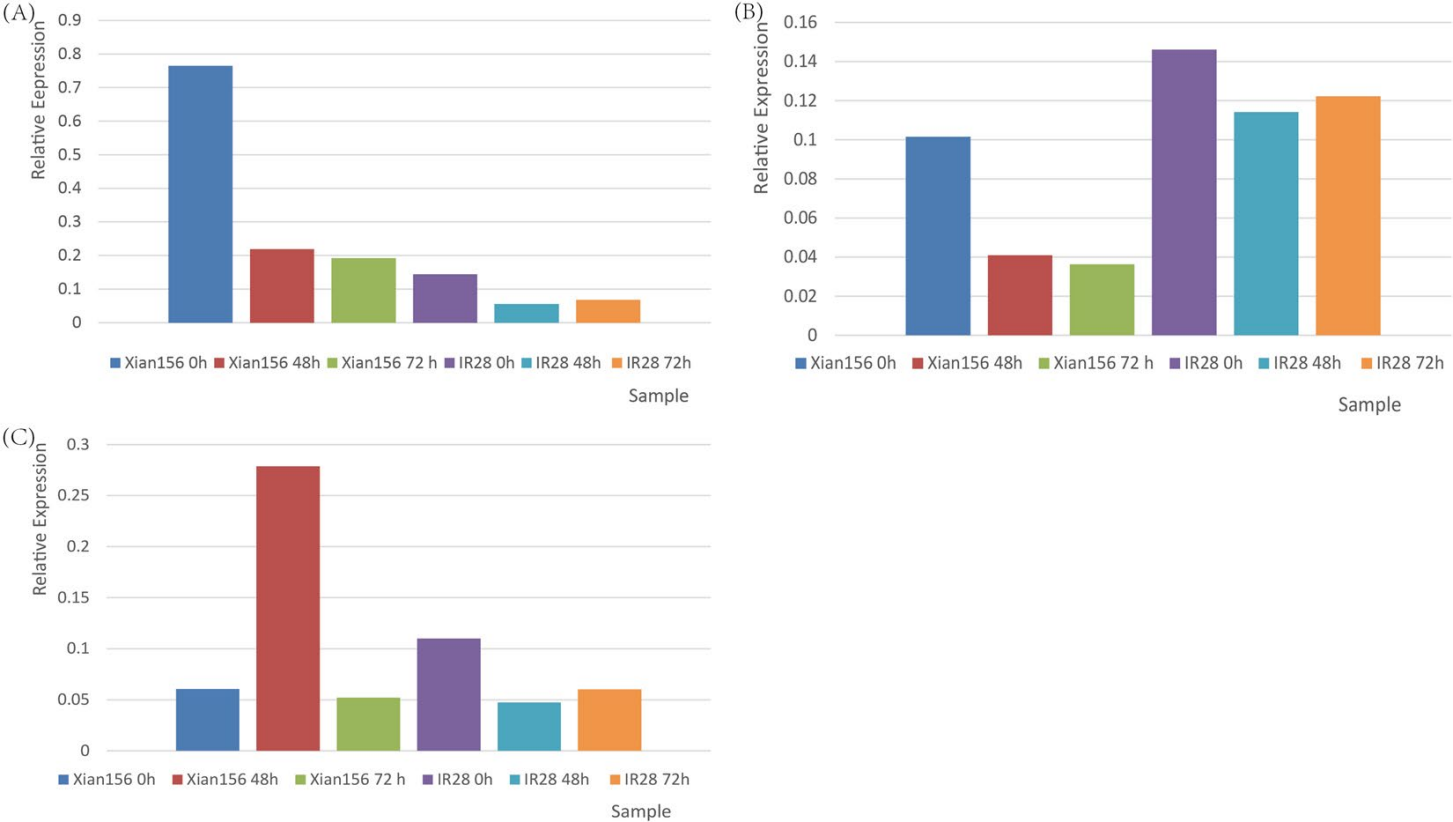
The expression of validated miRNAs by qPCR. The mRNA level was expressed relative to the Actin2 (BGIOSGA033259) expression level as 2^–ΔCt^. The (**A** and **B**) represent the qPCR data that came from the tolerant genotype Xian156 and sensitive genotype IR28, respectively.

To validate RNA-seq data, three genes were chosen for analysis by qPCR. The expression of BGIOSGA003836 and BGIOSGA021937 decreased sharply at 48 h after being subjected to salt stress, and then was kept steady at 72 h in both tolerant and sensitive cultivars. The expression pattern of the *TIFY 10* *A* gene (BGIOSGA031997) was different in these two genotypes, as it increased 3.6-fold at 48 h in the tolerant cultivar, whereas it decreased 0.57-fold in the sensitive cultivar. Then, the expression of BGIOSGA031997 decreased to around the expression of control at 72 h in a tolerant cultivar, whereas it increased slightly in the sensitive cultivar (Fig. 4).

### Identification of differentiated genes involved in the salt response

To explore the molecular mechanisms of a salt-tolerant genotype respond to salinity stress, differentiated expressed genes (DEGs) analysis was performed. The sequencing reads were mapped to the BGI rice reference genome, then the FPKM value of each gene and each transcript were calculated. For each time point in two genotypes, three biological replicates were used. Therefore, the p-value and False Discovery Rate (FDR) were calculated to measure the significance and reliability when taking all three biological replicates into account for reducing bias. In this study, the DEGs were selected when they met the criteria that FDR >0.1 and change fold >2 after eliminating bias with three biological replicates (Supplementary Table S5).

The number of up-regulated and down-regulated DEGs at different time points and between sensitive and tolerant rice genotypes are summarized in Table 3. At 48 h after Xian156 seedlings were exposed to salinity stress, and 1594 genes were found to be differentially expressed in Xian156 leaves, of which 440 up-regulated genes and 1154 down-regulated genes. The results showed that almost two times the number of genes were found differentially expressed at 72 h than at 48 h, and the up-regulated genes and down-regulated genes were almost equal. The comprehensive results showed that in salinity stress, more genes were down-regulated at 48 h and then genes were up-regulated, and down-regulated genes tend to reach almost equal status at 72 h. The comparison of DEGs at 48 h and 72 h showed that 2778 genes were differentially expressed at 48 h and 72 h. Under normal growth conditions, there were 1295 genes expressed and differentiated in Xian156 and IR28 leaves, of which 527 genes and 768 genes had more abundant expression in Xian156 and IR28 leaves, respectively. There were 1,196 genes that responded to salinity stress at 48 h, and almost two times more genes (total 3,308) started to respond to stress at 72 h, like the molecular response pattern in Xian156.

**Table 3:** The number of up-regulated and down-regulated DEGs at different time points between sensitive and tolerant rice genotypes.

DEG Comparison	All DEG	Up-regulated DEG	Down-regulated DEG
Tolerant, 0 h vs 48 h	1594	440	1154
Tolerant, 0 h vs 72 h	3285	1619	1666
Tolerant, 48 h vs 72 h	2778	1276	1502
Sensitive, 0 h vs 48 h	1196	487	709
Sensitive, 0 h vs 72 h	3308	1764	1544
Sensitive, 48 h vs 72 h	2419	1220	1199
0 h, Sensitive vs Tolerant	1295	768	527
48 h, Sensitive vs Tolerant	1072	516	556
72 h, Sensitive vs Tolerant	1494	759	735

**Figure 5:**
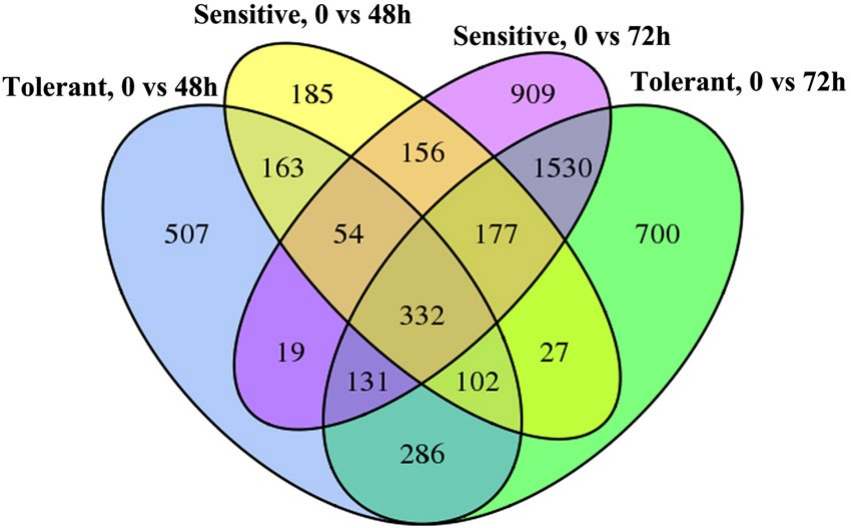
DEGs among time points after salinity stress application of two genotypes.

A total of 332 DEGs were found in the overlap regions of those four comparisons, which indicates these genes were differentially expressed at all timepoints regardless of genotype when subjected to salinity stress. There were 286 DEGs found at both 48 h and 72 h in the tolerant genotype, while 507 and 700 were only differentially expressed at 48 h and 72 h, respectively. Corresponding to the sensitive genotype, there were 156, 185 and 909 DEGs that were differentially expressed at two time-points at 48 h only and at 72 h only, respectively (Fig. 5).

### Annotation of DEGs response to salinity stress

The DEGs were assigned GO annotation after comparisons with Nr, Swiss-Prot, TrEMBL, KEGG and KOG databases as previously reported. In addition, the topGO tool (v2.26, http://www.bioconductor.org/packages/release/bioc/html/topGO.html) embedded in the R/Bioconductor package to normalize gene expression measurements, gene-wise correlation or differential expression analysis, enrichment analysis of GO terms, interpretation and visualization of the results^42^. In total, 8 pathways enriched with their KS value (significance) were less than e^−5^ (Table 4). These pathways were highly linked to the two-phase growth model in response to salinity in plants^4^. The rice seedlings primarily respond to the osmotic stress, which was associated with metabolism inhibition, such as cell expansion, cell wall synthesis, protein synthesis, stomata conductance, and photosynthetic activity after water interfered. Na^+^ from saline soil interfered with cationic sites involved in the binding of K^+^, calcium or magnesium^43^. Thus, the magnesium ion binding (GO:0000287) genes were differentially expressed under salinity stress. Cysteine proteinases of the clone *RD19* and *RD21* may be induced by changes in the osmotic potential of plant cells under high salt conditions^44,45^. This study supported the genes that encode cysteine proteinases could respond to water deficiency that was induced by salinity stress. The results also indicated that chromatin binding activity was also involved, which was possibly due to cell structure damage and repairs in response to osmatic stress. Previous work indicated that the later phase involved signaling and metabolic pathways for an oxidative burst because of the accumulation of ions. The ATP binding (GO:0005524) genes were associated with H^+^-ATPases, Ca^2+^-ATPases, Na^+^/K^+^-ATPases and ATP-binding cassette (ABC) transporters were involved in the later response to salinity stress. Some serine/threonine protein kinases could be activated by a salt-stress-elicited calcium signal, and then the results phosphorylate and activate various ion transporters, such as the plasma membrane Na^+^/H^+^ antiporter^46^. The ion accumulation also stimulated the production of reactive oxygen species (ROS). A total of 29 peroxidase activity (GO:0004601)-related genes that belonged to the ROS scavenging system were characterized as DEGs. We also found 44 and 84 DEGs had chromatin binding activity (GO:0003682) and heme binding (GO:0020037), respectively, which were a lack of reports and need further analysis to uncover their relationship with salinity stress.

**Table 4:** GO Classification of enriched DEGs by the topGO tool.

GO ID	GO Term	Annotated	Significant	KS
GO:0004601	peroxidase activity	259	29	1.7E-09
GO:0003682	chromatin binding	428	44	3.5E-07
GO:0005524	ATP binding	3813	366	1.7E-06
GO:0020037	heme binding	767	84	3.1E-06
GO:0016758	transferase activity, transferring hexosyl groups	715	68	4.7E-06
GO:0000287	magnesium ion binding	207	22	8.00E-06
GO:0004722	protein serine/threonine phosphatase activity	125	20	1.10E-05
GO:0004197	cysteine-type endopeptidase activity	46	8	1.20E-05

### DEGs at different time points in response to salinity stress

**Figure 6:**
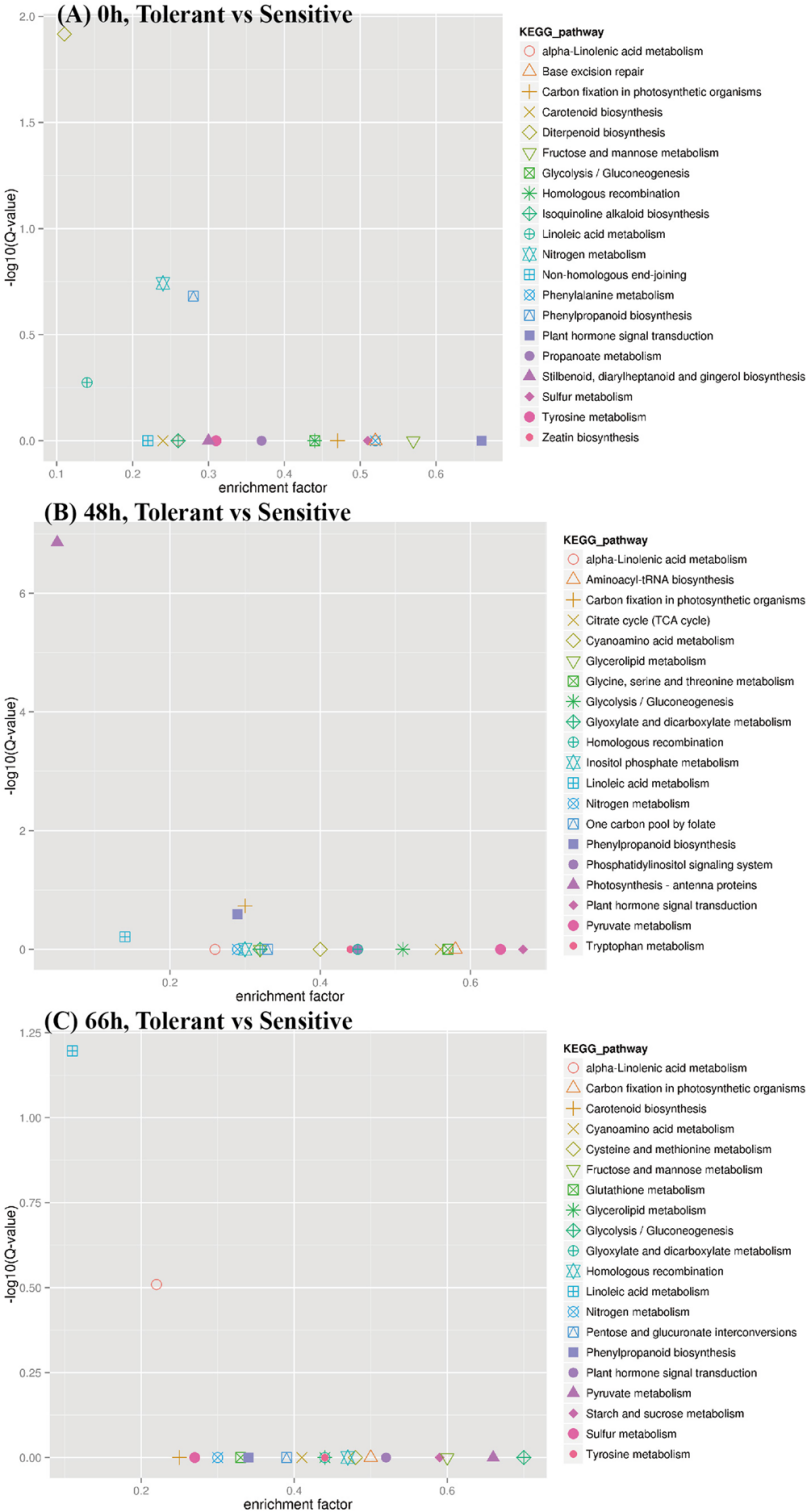
The KEGG enrichment of DEGs between the tolerant and sensitive rice genotypes.

To obtain insight into the gene functions and pathways, the DEGs between the tolerant and sensitive rice genotypes at 48 h and 72 h were analyzed using the KEGG database after enrichment analysis (Fig. 6). The horizontal axis showed an enrichment factor that indicated the ratio of number of DEGs out of all genes in a pathway, while the vertical axis illustrated the log(Q-value) and the Q-value represented the p-value for multiple hypothesis testing. The genes involved in linoleic acid metabolism, nitrogen metabolism, fructose and mannose metabolism, carotenoid biosynthesis, carbon fixation in photosynthetic organisms and glycolysis/gluconeogenesis pathways were differentially expressed between sensitive and tolerant genotypes at three time-points, which indicated these pathways were a natural difference between these two rice genotypes and might not be linked to salinity stress. The genes coding antenna proteins involved in photosynthesis were differentially expressed at 48 h but not at 0 h and 72 h. The glutathione metabolism, pyruvate metabolism, starch and sucrose metabolism and tyrosine metabolism were differentially expressed at 48 h and 72 h but not 0 h. This result illustrated that genes involved in those pathways were possibly the genes linked to salt tolerance.

### SNPs in transcription regions

To investigate the SNPs of these two genotypes and search possible SNPs that participate in their different responses to salinity stress, we compared the Xian156 and IR28 genotypes with the *indica* referent 93–11 genotype. The Xian156 genotype has a distant relationship with reference genotype 93–11, while its male parent IR24 has a close relationship with IR28. After applying a set of selection criteria, 148,286 SNPs were identified in 18 samples, including 99,045 in genic regions and 47,241 in intergenic regions (Supplementary Table S6). The distribution of SNPs on gene structures were also characterized. The transition type in each sample varied from 60.72% to 63.44%, which was a frequency higher than transversion type. In contrast, with the SNP number, distribution region and type, the heterozygosity in each sample varied substantially with a range from 21.71% to 62.13% (Supplementary Table S7).

**Figure 7:**
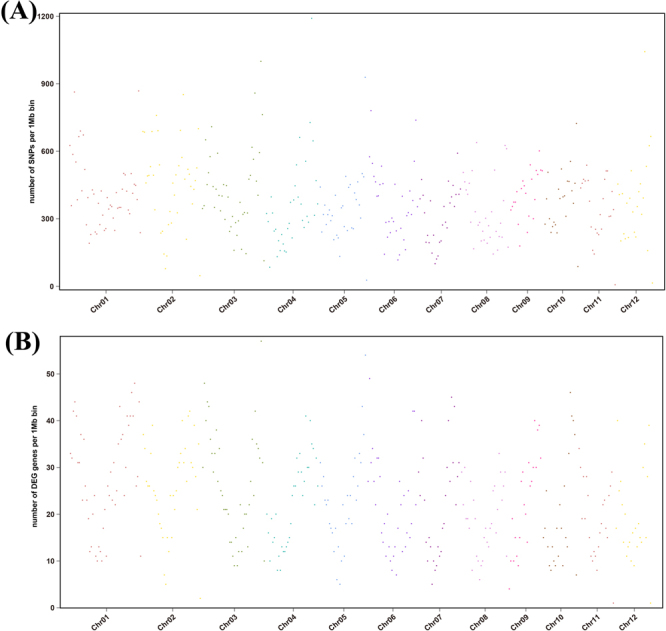
Genome-wide distributions of SNPs (**A**) and DEGs (**B**) in the rice genome. Number of SNPs and DEGs were calculated over non-overlapping 100 kb and 10 MB bins, respectively.

Additionally, we investigated the distribution of SNPs and DEGs to determine their correlation. Furthermore, we calculated the density of SNPs in non-overlapping 100 kb bins of each chromosome and found four significant peaks on chromosome 1 (Chr1: 41.3-41.4 Mb), chromosome 3 (Chr3: 1.4 Mb; 38.3-38.4 Mb), and chromosome 2 (Chr2: 10.1–10.2 Mb). We also calculated the density of DEGs in 10 Mb bins in the genome and found that some DEGs were located in SNP rich regions, such as chromosome 1 (Chr1: 30–40 Mb), chromosome 6 (Chr6: 0–10 Mb) and chromosome 9 (Chr9: 10–20 Mb) (Fig. 7). Additionally, We the SNPs and DEGs were both enriched in the central regions of chromosomes. The correlation illustrated that these SNPs could be linked to the tolerance of salinity stress in some regions of the rice chromosomes. These SNPs were useful as molecular markers for map-based cloning and functional characterization of genes that may be linked to the tolerance of salinity stress.

## Discussion

Genome-wide identification and functional prediction were also performed in various plants, including rice^47^, maize^48^, poplar^49^ and even the medical mushroom *Ganoderma lucidum*^50^. Salinity stress tolerance is a quantitative trait that is controlled by multiple genes^51^. The RNA-seq protocol is an effective tool for scanning the transcribed genes and their expression in a global picture, which was used to identify genes involved in the core salt-tolerance mechanisms in plants. In this study, we successfully used a total RNA-Seq approach to provide a powerful tool for in-depth exploration of its transcriptome. Starting from sequenced 18 RNA libraries, we identified more than 1,375 new genes as well as new gene structures with 148,286 SNPs. Cultivated rice has numerous wild relatives, and some of those relatives have been studied using high-throughput sequencing on their genome and transcriptome, including *Oryza rufipogon* Griff^16^. This is the first transcriptome report using the rice subspecies *indica* as the experimental material, which is also a valuable and widely distributed subspecies of rice. The high-throughput RNA-seq technology used in this study contributed to a better annotation of the rice genome. Furthermore, GO classification and functional analysis were carried out to obtain a better understanding of the functions of DEGs in response to salinity stress. These genes were mainly involved in hormone and calcium signaling pathways, transcription factors, ion metabolism and transfer, nitrogen metabolism and secondary metabolism, which were enriched in response to additional salt.

The first subgroup of salt-responsive genes was linked to plant hormone and calcium signaling pathways. After salt stress induction, a growth recovery phase followed an initial quiescence period, and these phases correlated with changes in the levels of the plant hormones abscisic acid (ABA), jasmonate (JA), gibberellic acid (GA), and brassinosteroid (BR)^52^. The expression of ABA-associated genes BGIOSGA008883, BGIOSGA023470 and BGIOSGA029635 increased under salinity stress, while the expression of BGIOSGA014475 and BGIOSGA025832 decreased. Furthermore, 64, 28 and 34 DEGs were linked to the jasmonate, gibberellic acid and brassinosteroid pathways. Previous studies suggest that Ca^2+^ plays an important role in the salt tolerance of plants. An additional level of regulation in calcium signaling is achieved via the action of calcium binding proteins, including CDPKs, CaMs, CMLs and CBLs. CBL proteins fulfill crucial functions in diverse Ca^2+^-dependent processes in plants. CBL-CIPK complexes are instrumental for relaying plant responses to many environmental signals and for regulating ion flux. The CBL-interacting protein kinase 9 in rice (BGIOSGA011542) maintained a stable level at 48 h, whereas it was up-regulated at 72 h in two genotypes, which showed that rice CBL9 is involved in the later phase of the response to salinity stress.

The second subgroup of salt-responsive genes were transcription factors. The TIFY gene family is a large group of transcription factors that are defined by a highly conserved TIFY motif (TIF[F/Y]XG). The previous report identified 20 TIFY genes in rice, and almost all the OsTIFY genes were responsive to one or more abiotic stresses, including drought, salinity, and low temperature. Additionally, 9 rice TIFY genes were responsive to jasmonic acid and wound treatments^53^. The expression of rice *TIFY 10* *A* (BGIOSGA031997) was stable after salinity treatment in sensitive IR28, whereas the expression of *TIFY 10* *A* increased approximately 3-fold at 48 h and was maintained at high level at 72 h in tolerant Xian156. That outcome indicated this TIFY 10 A possibly had a positive correlation with the salinity tolerance of Xian 156.

The third subgroup of DEGs was highly associated with ion metabolism and transfer. Several stress-inducible genes were significantly up-regulated or down-regulated after salinity treatment. The salinity stress could induce osmotic stress in roots where the membrane plays an important role in regulation of Na^+^ ion homeostasis. An uncharacterized stress-inducible membrane pore gene (BGIOSGA001811) was expressed very low under normal conditions, while its FPKM value increased to approximately 1.0 in the sensitive genotype IR28 and increased to approximately 2.9 in the tolerant genotype Xian156 under salinity stress.

The nitrogen metabolism pathway was identified as the fourth subgroup of salt-responsive genes among three time-points both in the tolerant and sensitive rice genotypes using KEGG enrichment analysis (Supplementary Figure S5). Interestingly, the expression of genes in the nitrogen metabolism pathway showed no difference at 0 h and then were differentially expressed after salinity stress application in both the sensitive and tolerant genotypes. The genes in nitrogen metabolism pathways were maintained at a steady level in the early phase of salinity stress, whereas they were differentially expressed at a later phase. BGIOSGA007024 (Name in NCBI: OsI_06091) was annotated as a transferase involved in the glutamine metabolic process (GO:0006541) in mitochondria. The chloroplastic glutamine synthetase (GS2) genes that were overexpressed in transgenic rice could enhance salt tolerance^54^. Among these DEGs only in the tolerant genotype, 13 were newly identified genes. The GO annotation showed that plastids were highly linked to the tolerant genotype because 6 genes were found in the plastid. Plant glutathione transferases (GSTs) are induced in various biotic and abiotic stresses, and they are important for protecting plants against oxidative damage^55^. Five GSTs in leafy spurge plants showed differential expression patterns when exposed to diclofop-methyl, cold and drought stress^56^. The glutathione S-transferase GSTF1 in sensitive IR28 was not significantly expressed before or after salinity treatment. In contrast, the expression of GSTF1 almost doubled at 48 h and then doubled again at 72 h, which indicated that the expression patterns in Xian156 and IR28 were significantly different.

The salinity stress also influenced the biosynthesis of secondary metabolites, such as stilbenoids, diarylheptanoids, gingerols and flavonoids. The KOG analysis showed at least 101 rice genes were associated with secondary metabolite biosynthesis, transport and catabolism, of which 27 DEGs were linked to flavonoid biosynthesis. The RNA-seq results showed that gene BGIOSGA005546, which is involved in the biosynthetic process, decreased by almost 0.3-fold at 48 h both in the sensitive and tolerant rice genotypes, and the expression level was steady compared to control. In contrast, BGIOSGA019338 increased by almost 2-fold at 48 h and was kept steady, which showed a different expression pattern. The annotation showed that BGIOSGA019338 played an important role in signaling and regulation, which was a response to salinity stress, fructose and cadmium ion, and the gene was also involved in the flavonoid biosynthetic process, water transport and cellular copper ion homeostasis. BGIOSGA005362 could response to sucrose and UV-B in addition to being involved in the flavonoid biosynthetic process. This gene changed in response to salinity stress in the late phase by dramatically increasing by approximately 7-fold at 72 h.

In summary, our research sheds light on the salinity stress-mediated signal transduction pathways during salinity stress acclimation. The identified differentially expressed genes could also provide references for selecting salt-tolerant breeding materials in rice pre-breeding processes.

## Electronic supplementary material

Supplementary Figures S1 to S5

Supplementary Table S1

Supplementary Table S2

Supplementary Table S3

Supplementary Table S4

Supplementary Table S5

Supplementary Table S6

Supplementary Table S7
