# Supplementary material for: Comparative transcriptome analysis reveals molecular response to salinity stress of salt-tolerant and sensitive genotypes of indica rice at seedling stage
Source: Sci Rep. 2018 Feb 1;8:2085. doi: 10.1038/s41598-018-19984-w (PMC5794784; doi:10.1038/s41598-018-19984-w)

Comparative transcriptome analysis reveals molecular response to salinity stress of salt-tolerant and sensitive genotypes of *indica* rice at seedling stage

Jun Wang[1, 2], Jinyan Zhu[1, 2], Yadong Zhang [1],Fangjun Fan[1, 2], Wenqi Li[1, 2], Fangquan Wang[1, 2],Weigong Zhong[1, 2], Cailin Wang [1], Jie Yang [1, 2]

1. Institute of Food Crops, Jiangsu Academy of Agricultural Sciences/Nanjing Branch of Chinese National Center for Rice Improvement, Nanjing 210014, China；2. Jiangsu Co-Innovation Center for Modern Production Technology of Grain Crops, Yangzhou 225009, China

Correspondence and requests for materials should be addressed to J.Y. (email:yangjie168@aliyun.com)

Supplementary Figures

Figure S1. The growth vigor of these two genotypes in the absence of salinity. The leaf four rows were tolerant Xian156 seedlings, while right four rows were sensitive IR28 seedlings.

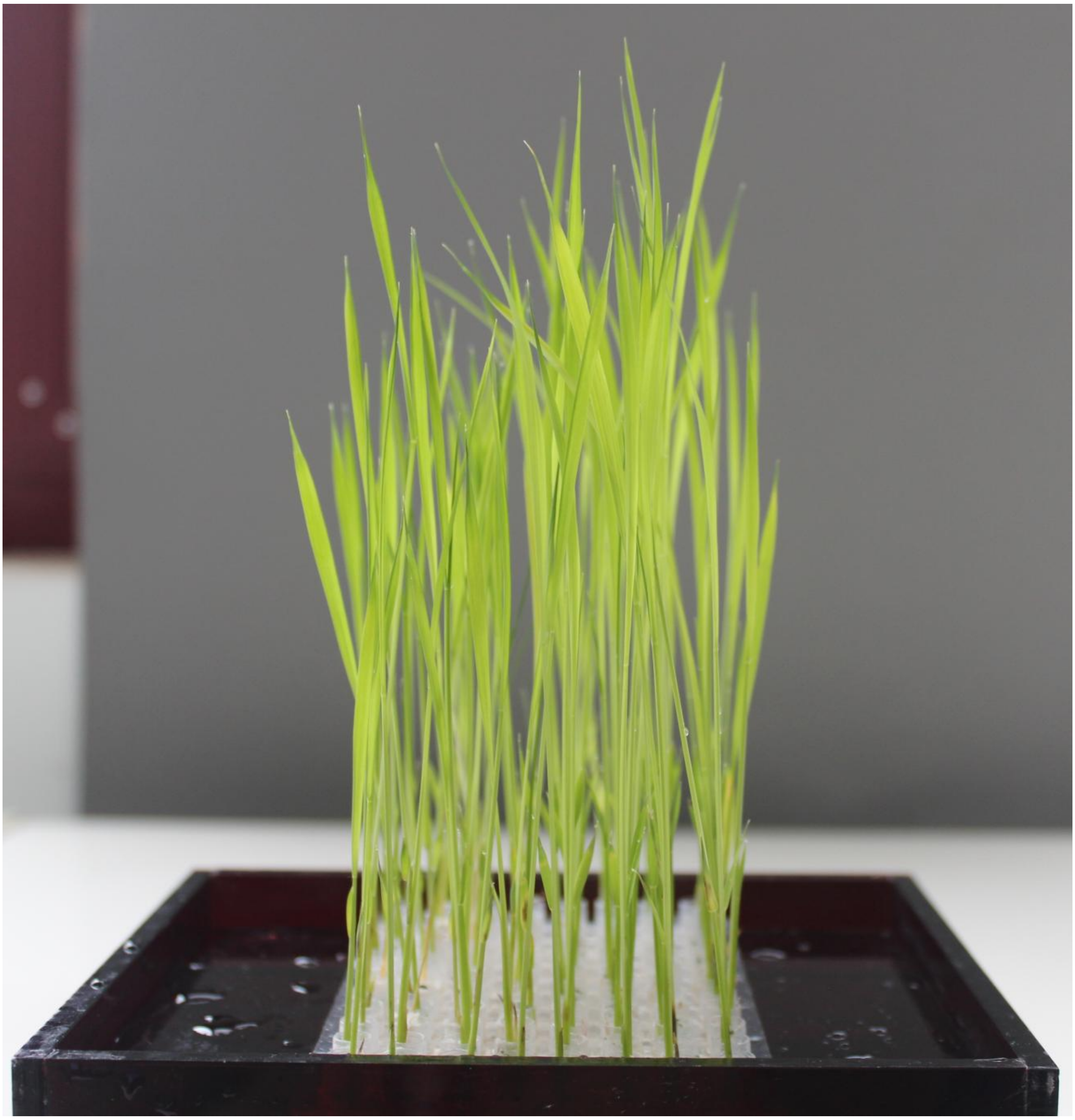

Figure S2. The saturation curve of all sequencing data from 18 RNA library indicated that sequencing depth was enough for transcriptome assembly and expression detection.

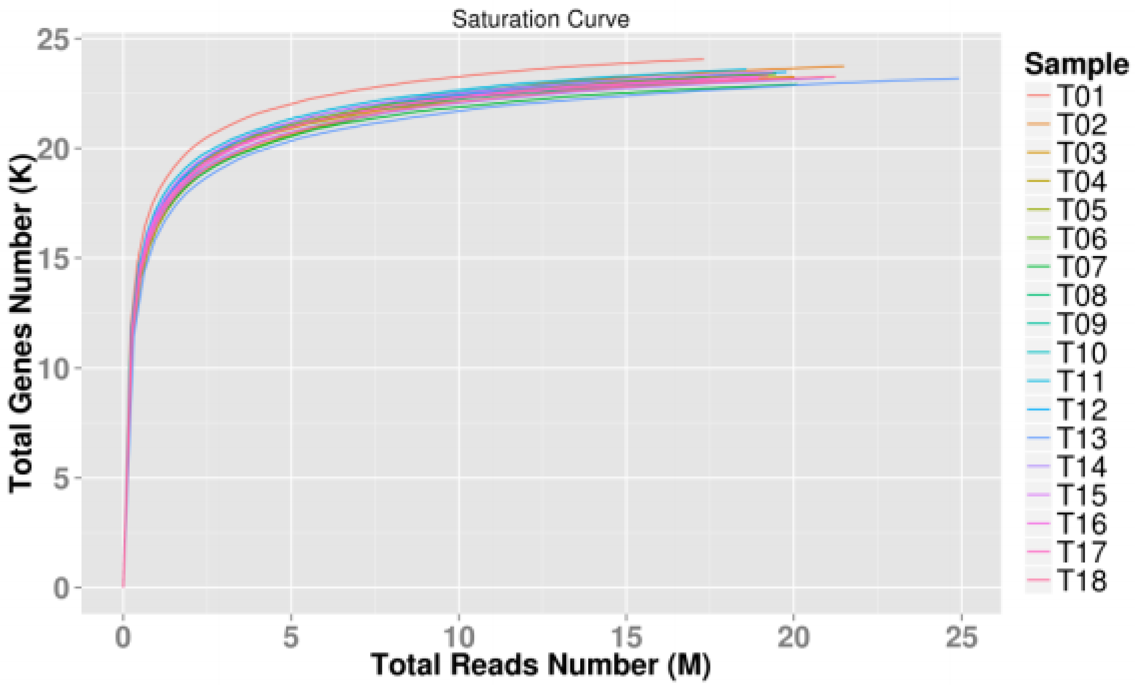

Figure S3. Venn diagram of lncRNAs predicted by CPC, CNCI, CPAT and Pfam pipelines.

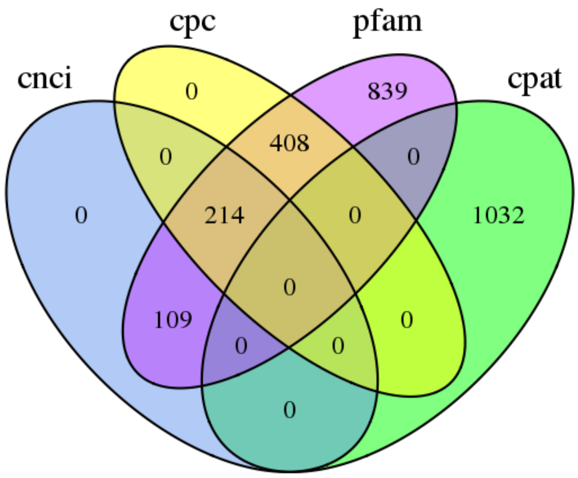

Figure S4. After comparing with proteins in NCBI NR database, homologs of most newly identified genes were also found in other monocots, such as *Oryza brachyantha*, *Zea mays*, *Oryza sativa* and *Setariz italic*.

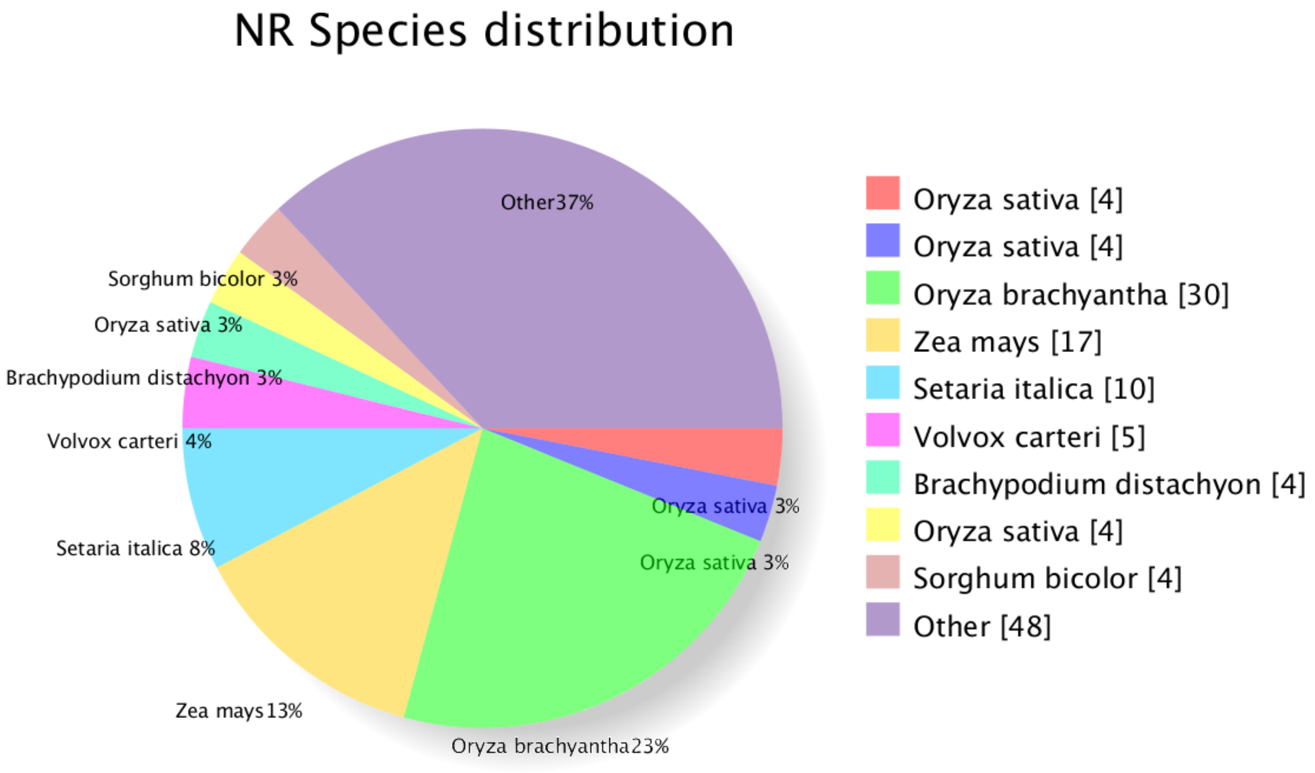

Figure S5. The KEGG enrichment of DEGs between different time-points.

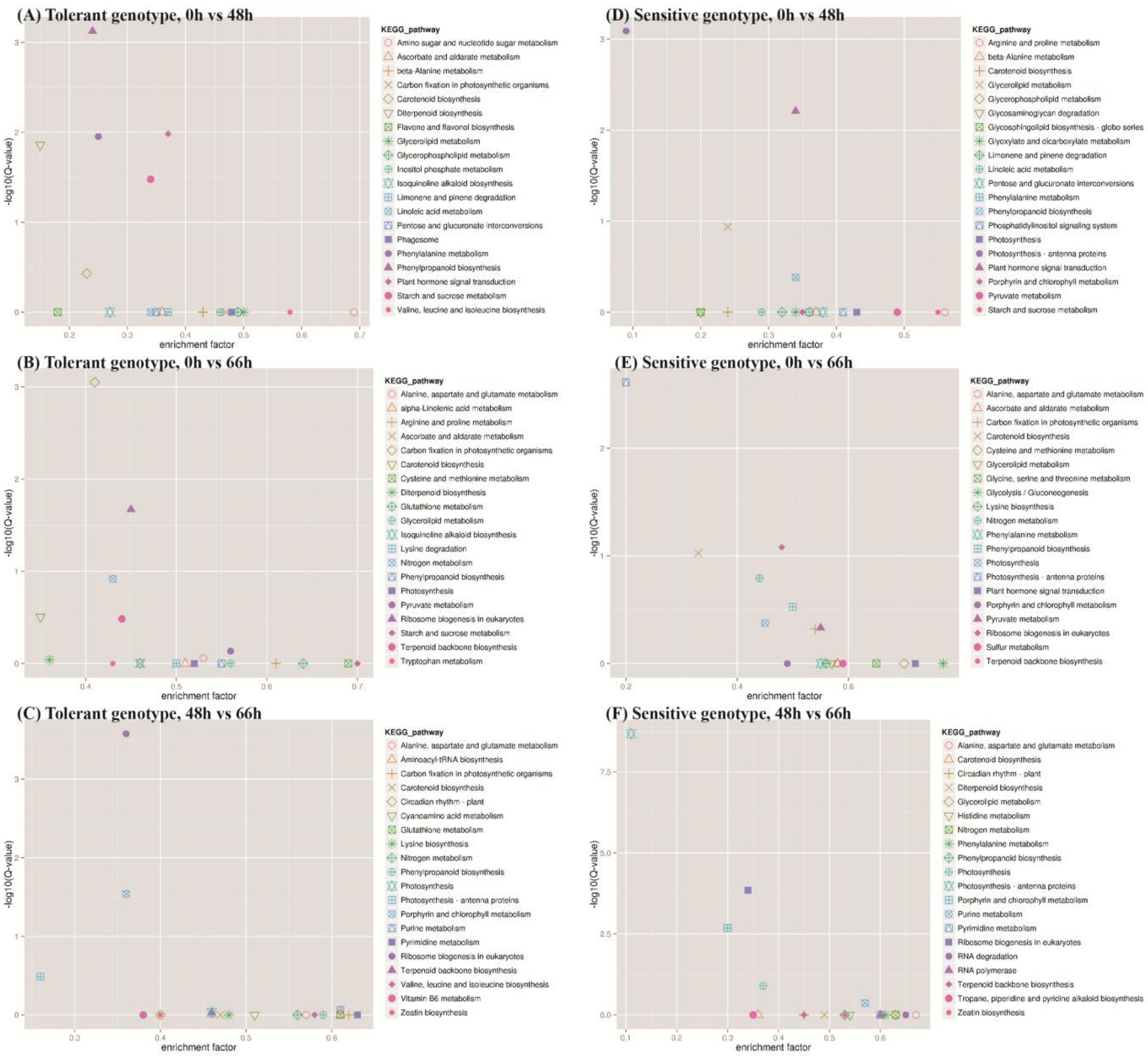

Supplement: Supplementary file 1 — Supplementary Figures S1 to S5 [file 41598_2018_19984_MOESM1_ESM.pdf]
